# Hereditary Transthyretin Amyloidosis in Israel: Genetic Landscape and Clinical Characteristics

**DOI:** 10.1111/ene.70057

**Published:** 2025-01-29

**Authors:** Amir Dori, Odelia Chorin, Noa Ruhrman‐Shahar, Avi Fellner, Tayir Alon, Haike Reznik‐Wolf, Ortal Barel, Dana Fourey, Osnat Itzhaki Ben Zadok, Yaron Aviv, Vera Nikitin, Merav Ben‐David, Efrat Shavit‐Stein, Rivka Goldis, Batia Kaplan, Daniela Shapiro, Elon Pras, Arthur Pollak, Vardiella Meiner, Michael Arad, Lior Greenbaum

**Affiliations:** ^1^ Department of Neurology Sheba Medical Center Ramat Gan Israel; ^2^ Faculty of Medical & Health Sciences Tel Aviv University Tel Aviv Israel; ^3^ The Danek Gertner Institute of Human Genetics Sheba Medical Center Ramat Gan Israel; ^4^ Raphael Recanati Genetic Institute Rabin Medical Center Petah Tikva Israel; ^5^ Department of Neurology Rabin Medical Center Petah Tikva Israel; ^6^ Genomic Unit, Sheba Cancer Research Center Sheba Medical Center Ramat Gan Israel; ^7^ The Leviev Heart Center Sheba Medical Center Ramat Gan Israel; ^8^ Cardiovascular Department Rabin Medical Center Petah Tikva Israel; ^9^ Institute of Hematology Sheba Medical Center Ramat Gan Israel; ^10^ Department of Cardiology Hadassah Medical Center, Hebrew University of Jerusalem Jerusalem Israel; ^11^ Department of Genetics Hadassah Medical Center, Hebrew University of Jerusalem Jerusalem Israel

**Keywords:** cardiomyopathy, hereditary amyloidosis, polyneuropathy, transthyretin, *TTR*

## Abstract

**Background:**

Hereditary transthyretin (ATTRv) amyloidosis is a rare, adult‐onset autosomal‐dominant disorder caused by pathogenic variants in the transthyretin (*TTR*) gene. Data about relevant variants in specific populations and typical initial manifestations may facilitate early diagnosis and treatment. We here describe the genetic landscape of ATTRv amyloidosis in Israel.

**Methods:**

Genetic and clinical data of *TTR* variant carriers and ATTRv amyloidosis patients were collected from a national referral clinic and other subspecialty clinics in Israel. Genotype–phenotype correlations of the detected variants were detailed. In addition, two large Israeli exome sequence (ES) databases were screened for *TTR* variants.

**Results:**

Seven heterozygous disease‐causing variants in *TTR* were identified among 95 adults (52 males, 50.7%). The Ser77Tyr variant was found in 68 (71.6%) subjects of Jewish Yemenite ancestry. Val122Ile was found in 9 (9.4%) subjects and was the only variant detected in individuals of Arab ethnicity. Other variants were Thr60Ala, Val30Met, Val32Ala, Ala81Val, and Glu89Val. Thirty‐five individuals were ATTRv amyloidosis patients (25 males, 71.4%), diagnosed at a mean age of 62.5 ± 6.7 years, and 23 (63.7%) were due to Ser77Tyr. Initial symptoms were mostly related to carpal tunnel syndrome, and the sensitivity of scintigraphy was low for Ser77Tyr but high for Thr60Ala and Val32Ala variants. *TTR* pathogenic variants were detected in 14 of approximately 36,600 subjects who underwent ES, including Val122Ile in 9 subjects of Arab ethnicity.

**Conclusions:**

Most ATTRv amyloidosis cases in Israel are attributable to the Ser77Tyr variant. However, other variants also contribute to disease occurrence, and testing is warranted in clinically suspected patients.

## 1 Introduction

Hereditary transthyretin (ATTRv) amyloidosis is a rare late‐onset multisystemic disorder, attributed to disease‐causing variants in the transthyretin (*TTR*) gene and inherited in an autosomal dominant manner [1, 2]. The main clinical features include a combination of progressive peripheral and autonomic neuropathy, cardiomyopathy, and involvement of other body systems, such as gastrointestinal, ocular, renal, and brain meninges [3, 4]. The presence of the above features in pathogenic/likely‐pathogenic variant (henceforth referred to as variant) carriers mandates timely investigation to confirm amyloid infiltration in tissue and early initiation of treatment, such as TTR stabilizers or RNA silencers [5, 6]. However, in the absence of a positive family history, early isolated sensory, autonomic, or cardiac symptoms are typically attributed to various other common disorders.

More than 130 variants in *TTR* have been described [7], with substantial differences in their association with age of disease onset, penetrance, and clinical course [8]. Some variants cause predominantly neurologic symptoms, while others cause predominantly cardiac or mixed manifestations [9, 10].

Several *TTR* variants are more frequently identified in specific ethnic origins [11]. Their recognition may accelerate the diagnosis of ATTRv amyloidosis and facilitate the screening of at‐risk family members. In Israel, attention has been given to the Ser77Tyr variant among individuals of Jewish Yemenite descent. We previously described 49 subjects with this heterozygous variant in detail, focusing on initial presentation and diagnostic features [12]. We estimated that in this population, complete penetrance of the disease occurs at approximately age 70 years. However, a few other *TTR* variants were also reported in Israel [13]. Lack of awareness of these variants' occurrence may delay diagnosis and treatment, which is essential for improved prognosis [14, 15].

The current study describes the genetic landscape of *TTR* variants in Israel, among Jewish and Arab populations. We describe the main neurologic and cardiac features of the frequent variants. The source data is based on a national referral center and a survey of other clinics in Israel. We also screened exome sequencing (ES) databases representing the Israeli population to detect variants in *TTR*.

## 2 Methods

We retrospectively reviewed the medical files of adult (age 18 years and above) ATTRv amyloidosis patients and other carriers of variants in *TTR*, evaluated between January 2016 and August 2024 at the neuromuscular clinic in Sheba Medical Center (SMC), Israel. The Israeli Ministry of Health recognized this clinic in January 2023 as a national referral center for ATTRv‐polyneuropathy (ATTRv‐PN). Additionally, several other major Israeli cardiology, neurology, and genetic clinics/institutes were contacted to retrieve data on additional carriers of *TTR* variants, with and without a diagnosis of ATTRv‐amyloidosis. The SMC institutional review board approved this study.

All participants underwent genetic screening for *TTR* variants. In some cases, the entire coding region of *TTR* was sequenced. In other cases, targeted Sanger sequencing of a specific variant in *TTR* was undertaken according to reported ancestry and/or family medical history. Testing was performed in commercial laboratories or genetic institutes in Israel and abroad. Demographic and clinical data were retrieved from medical records to obtain information regarding sensory, motor, autonomic, and cardiac‐related signs and symptoms. All participants completed a detailed neurological examination. We documented the age at onset of initial symptoms and the sequence of ATTRv‐amyloidosis‐related symptoms. Symptoms related to ATTRv‐PN included carpal tunnel syndrome (CTS), manifestations of length‐dependent sensory or sensory‐motor polyneuropathy, muscle weakness, and autonomic dysfunction (orthostasis and, in males, erectile dysfunction). Dyspnea was considered a cardiac‐related symptom, and gastrointestinal involvement was related to the appearance of frequent diarrhea or constipation, vomiting, or weight loss. Ocular symptoms were not systematically recorded. Leptomeningeal manifestations were absent in our population.

Patients were asked to complete a laboratory evaluation for systemic disorders that cause neuropathy, and data about renal failure, diabetes mellitus/pre‐diabetes, and monoclonal gammopathy of undetermined significance (MGUS) were obtained. Information regarding disease treatment modalities was collected, including liver transplantation and the use of the available disease‐modifying drugs Tafamidis and Patisiran.

Nerve conduction studies (NCS) and electromyography (EMG) were performed to determine the presence of large fiber polyneuropathy and denervation. The sympathetic skin response (SSR) was tested in the palm and foot on one side when the sural study was normal. A skin biopsy from the distal leg was used to diagnose small fiber neuropathy, determined by epidermal nerve fiber density (ENFD) below the 5th percentile per age.

Cardiac evaluation included 12‐lead electrocardiography and transthoracic echocardiography. When left ventricular global longitudinal strain (GLS) measurement was performed, an absolute value < 15 was considered abnormal. Cardiac scintigraphy with single photon emission computed tomography (SPECT) in the majority of cases employed ^99m^Tc‐pyrophosphate (PYP) or ^99m^Tc‐3,3‐diphosphono‐1,2‐propanodicarboxylic acid (DPD) and was considered positive for ATTRv‐cardiac amyloidosis (ATTRv‐CM) when the Perugini grade was ≥ 2 [16] and a monoclonal gammopathy was excluded. Cardiac magnetic resonance imaging (CMR) was determined to be abnormal when typical late gadolinium enhancement (LGE) was evident [17].

Amyloid infiltration was determined by tissue biopsy analysis to identify Congo red positive deposits, obtained mainly from the skin. In a few cases, biopsies were taken from the endomyocardium, abdominal fat, sural nerve, gastrointestinal mucosa, or bone marrow. In cases with co‐occurring clonal plasma cell dyscrasia, amyloid typing was performed by western blot analysis to exclude amyloid light‐chain (AL) amyloidosis [18, 19].

A diagnosis of ATTRv‐amyloidosis was determined in subjects with a *TTR* disease‐causing variant and a positive biopsy. When biopsies were not available or negative for Congo red staining, cardiac uptake on scintigraphy was defined as consistent with histologically proven amyloid deposition if plasma cell dyscrasia was negative. Age at diagnosis was determined according to the positive biopsy or scintigraphy (the earliest). In one case, both biopsy and scintigraphy were not available, and amyloid infiltration was based on typical CMR imaging [16, 20, 21].

In the presence of amyloid infiltration, ATTRv‐PN was defined by evidence of large or small fiber polyneuropathy, and ATTRv‐CM by increased thickness of the interventricular septum wall (IVS) diameter ≥ 12 mm according to transthoracic echocardiography, positive cardiac scintigraphy, or typical CMR imaging [16]. The absence of cardiac symptoms of dyspnea did not exclude the ATTRv‐CM diagnosis.

To estimate the frequency of a *TTR* variant among the Israeli population, we retrieved data about all pathogenic (P) or likely pathogenic (LP) *TTR* variants from ES databases of two large medical centers in Israel: SMC and Hadassah Medical Center (HMC). ES was performed for adult and pediatric subjects due to multiple indications, and healthy participants were also included. Variant classification was based on the American College of Medical Genetics and Genomics (ACMG) recommendations [22]. The available data regarding identified subjects in the ES databases included sex, ancestry, and the indication for ES.

Last, unrelated controls of Jewish Yemenite and Iranian descent were screened for the Ser77Tyr and Val32Ala variants, respectively, as these populations were under‐represented in the ES databases. These individuals underwent prenatal carrier screening at SMC Genetic Institute, and their medical histories were unavailable.

## 3 Results

### 3.1 Epidemiology of TTR Variants in Israel

**FIGURE 1:**
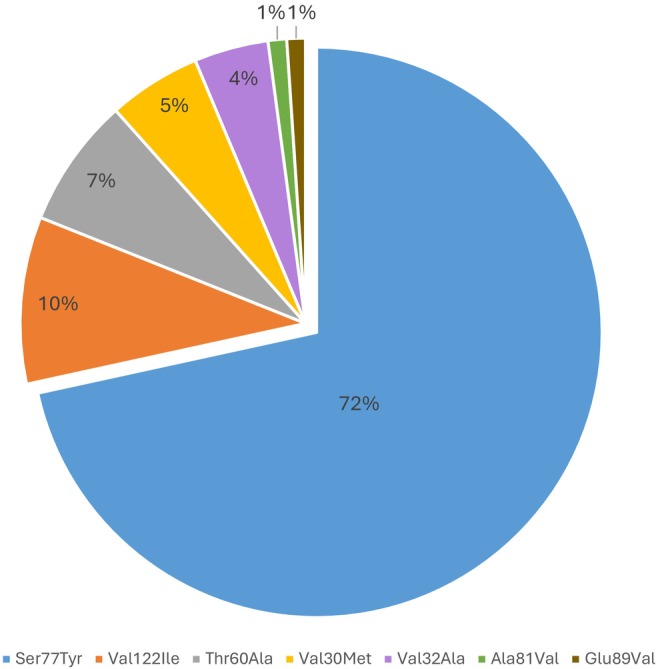
The proportion of *TTR* variants in clinically evaluated participants.

We identified 95 subjects (52 males, 54.7%) with one of seven heterozygous *TTR* variants (Table 1, Figure 1). The most frequently identified variant was Ser77Tyr (p.Ser97Tyr), detected in 68 Jewish subjects of Yemenite descent from 8 families. Forty‐nine of them, including 19 patients and 30 carriers, were previously described in detail [12].

**TABLE 1:** Disease‐causing variants in *TTR*, identified among 95 clinically evaluated participants.

Variant (*)	Subjects with the variant		Families	Origin	ATTRv amyloidosis patients			
	*n*	Male, *n* (%)	*n*	Country and/or Ethnicity	*n*	Male, *n* (%)	Alive at clinical assessment	Main phenotype
Ser77Tyr (c.290C>A,p.Ser97Tyr)	68	35 (51.5)	8	Yemen (J)	23	16 (69.6)	18	N
Val122Ile (c.424G>A, p.Val142Ile)	9	7 (77.8)	7	Muslim Arab‐3, African‐3, Ashkenazi (J)‐2, Yemen/Iran (J)‐1	1	1 (100.0)	1	C
Thr60Ala (c.238A>G, p.Thr80Ala)	7	5 (71.4)	1	Iraq (J)	5	4 (80.0)	3	M
Val30Met (c.148G>A, p.Val50Met)	5	2 (40.0)	3	Tunisia (J)‐1, Ashkenazi (J)‐4	1	1 (100.0)	0	N
Val32Ala (c.155 T>C, p.Val52Ala)	4	2 (50.0)	1	Iran (J)	3	2 (66.7)	3	M
Ala81Val (c.302C>T, p.Ala101Val)	1	0	1	Ashkenazi (J)	1	0	1	C
Glu89Val (c.326A>T, p.Glu109Val)	1	1 (100.0)	1	Ashkenazi (J)	1	1 (100.0)	1	C
Total	95	52 (54.7)	22		35	25 (71.4)	27	

Abbreviations: ATTRv amyloidosis, hereditary transthyretin amyloidosis; C, cardiac; J, Jewish; M, mixed; *n*, number; N, neurologic. (*) Annotated according to transcript NM_000371.4.

The Val122Ile (p.Val142Ile) variant was identified in nine subjects. Three of them were of Muslim Arab origin from two families, and this was the only variant detected in this population. Three subjects were immigrants from Africa, consistent with previous reports about the origin of this variant [23]. Nevertheless, it was also detected in two Jewish Ashkenazi subjects (father and son) and in one individual of mixed Yemenite and Iranian origin.

The Thr60Ala (p.Thr80Ala) was the third most common variant, detected in seven subjects, all from a large Jewish family of Iraqi descent.

Five individuals from three unrelated Jewish families, two of Ashkenazi ancestry and one originally from Tunisia, carried the Val30Met (p.Val50Met) variant. The Val32Ala (p.Val52Ala) variant, which was previously reported in a single Jewish Iranian patient in Israel [24] was identified in four of her family members.

Two variants, Ala81Val (p.Ala101Val) and Glu89Val (p.Glu109Val) were identified, each in a single subject. Both Ala81Val [7, 25, 26] and Glu89Val [27] were previously reported as disease‐causing variants.

All seven *TTR* variants above were classified as pathogenic/likely pathogenic according to the ACMG criteria [22] and consistent with a diagnosis of ATTRv amyloidosis (as detailed below).

Although most carriers were identified and evaluated at the national ATTRv clinic in SMC, their residences were distributed throughout Israel.

### 3.2 Diagnostic Characteristics of Patients With Confirmed ATTRv Amyloidosis

A total of 35 subjects (25 males, 71.4%) were diagnosed as ATTRv amyloidosis patients. The evolution of symptoms varied according to the main identified variants, although initial symptoms were most commonly related to CTS in 31 patients, occurring at an average age of 57.8 ± 7.0 years (range 43–75). Symptoms related to polyneuropathy, such as sensory disturbance in the lower limbs, followed at an average age of 60.2 ± 7.3 years (range 45–75) in 27 patients.

The diagnosis was confirmed by detecting amyloid in a biopsy in 29 of 30 (96.7%) of cases that performed this procedure at a mean age of 61.4 ± 6.4 years (range 43–73), and/or scintigraphy in 13 of 21 (61.9%) cases, at a mean age of 64.4 ± 5.9 years (range 57–75). Amyloid was detected in 26 of 28 (92.9%) of skin, 3 of 4 (75%) endomyocardial, 1 of 2 (50%) bone marrow, and in one rectal colon biopsy. In three cases, an abdominal fat biopsy was negative and more than one organ was sampled in seven cases. At the time of diagnosis, at a mean age of 62.5 ± 6.7 years (range 43–75), 30 of 35 (85.7%) patients had small and/or large fiber polyneuropathy. In 20 of 35 (57.1%) cases, a large fiber, and in 23 of 28 (82.1%), a small fiber neuropathy was identified, and in only one case, large fiber neuropathy was not accompanied by small fiber loss in the skin biopsy. Echocardiography showed an increased IVS thickness in 25 of 35 (71.4%) cases, GLS was abnormal in 16 of 20 (80%) and CMR showed an amyloidosis‐compatible LGE pattern in 14 of 16 (87.5%) cases. At diagnosis, symptoms of dyspnea were reported by 22 and gastrointestinal‐related symptoms in seven cases.

Systemic disorders that may cause or worsen polyneuropathy that were identified included pre‐diabetes or type 2 diabetes mellitus (T2D) in 14, and chronic renal failure and MGUS each in four. The diagnostic and genotype–phenotype characteristics of the common variants are detailed in Table 2 and in the supplementary data.

**TABLE 2:** Genotype–phenotype correlation and diagnostic characteristics of the three most common *TTR* variants in patients.

*TTR* variant	Ser77Tyr	Thr60Ala	Val32Ala
Number of patients	23	5	3
Males, *n* (%)	16 (69.6)	4 (80.0)	2 (66.6)
Age at symptom onset, years ± SD			
Carpal tunnel syndrome	57.7 ± 5.8	52.9 ± 6.2	57.2 ± 5.7
Polyneuropathy	59.7 ± 5.8	56.7 ± 12.8	58.2 ± 4.8
Symptoms at diagnosis, *n*/total (%)			
Age at diagnosis	61.9 ± 5.3	61.0 ± 12.2	59.9 ± 3.8
Carpal tunnel syndrome	20/23 (87.0)	5/5 (100)	3/3 (100)
Length‐dependent polyneuropathy	18/23 (78.3)	3/5 (60)	3/3 (100)
Autonomic	11/23 (47.8)	3/5 (60)	2/3 (67.7)
Lower extremity weakness	14/23 (60.9)	2/5 (40)	2/3 (67.7)
Dyspnea	17/23 (73.9)	4/5 (80)	1/3 (33.3)
Gastrointestinal related	5/23 (21.7)	1/5 (20)	1/3 (33.3)
Diagnostic characteristics, *n*/total (%)			
Main phenotype	Neurologic	Cardiac	Mixed
Positive biopsy	21/21 (100.0)	3/4 (75.0)	3/3 (100.0)
Large fiber neuropathy	16/23 (69.6)	1/5 (20.0)	1/3 (33.3)
Small fiber neuropathy	17/19 (89.5)	2/4 (50.0)	2/3 (67.7)
NYHA stage	2.1 ± 1.2	2.4 ± 0.9	1.3 ± 0.6
IVSd ≥ 12 mm	18/23 (78.3)	3/5 (60.0)	0/3 (0.0)
GLS < |−15|	14/16 (87.5)	1/2 (50.0)	1/3 (33.3)
Scintigraphy Perugini grade ≥ 2	3/10 (30.0)	4/5 (80.0)	3/3 (100.0)
CMR with amyloid‐compatible LGE	11/12 (91.7)	1/1 (100.0)	0/1 (0.0)
Concurrent systemic disease at diagnosis			
Diabetes mellitus/prediabetes	13/23 (56.5)	0/5 (0.0)	1/3 (33.3)
Chronic renal failure	3/23 (13.0)	1/5 (20)	0/3 (0.0)
MGUS	4/23 (17.4)	0/5 (0.0)	0/3 (0.0)

Abbreviations: CMR, cardiac magnetic resonance; GLS, global longitudinal strain; IVSd, interventricular septum diameter; LGE, late gadolinium enhancement; MGUS, monoclonal gammopathy of undetermined significance; *n*, number of patients who performed the test; NYHA, New York Heart Association.

Liver transplantation was performed in two cases. Tafamidis and Patisiran became available in Israel in 2016 and 2019, respectively. Since then, 23 patients have been treated with Tafamidis and/or 17 with Patisiran.

At the time of manuscript preparation, 27 patients were alive, consistent with a disease prevalence of 2.7 per million in the Israeli population, which is approximately 10 million, according to the Israeli Central Bureau of Statistics in 2024 [28].

### 3.3 Characteristics of TTR Variant Carriers

Sixty individuals (27 males, 45%) were heterozygous carriers of one of the five *TTR* variants but did not fulfill the diagnostic criteria for ATTRv amyloidosis: Ser77Tyr (*n* = 45), Val122Ile (*n* = 8), The60Ala (*n* = 2), Val30Met (*n* = 4), or Val32Ala (*n* = 1). At evaluation, at a mean age of 41.6 ± 12.4 years (range 18–79), 25 of 60 (41.6%) of these carriers reported various sensory symptoms in the hands and 12 of 60 (20.0%) in the feet. NCS and EMG at a mean age of 41.5 ± 12.3 years (range 18–79) showed evidence for a median neuropathy supporting CTS in 14 of 49 (28.6%) cases, which was bilateral in 11 cases.

In 5 of 45 (11.1%), the SSR was abnormal at the foot, but none of the carriers showed evidence of a large fiber polyneuropathy or muscle denervation. Skin biopsy was performed at a mean age of 42.5 ± 13.0 years (range 18–77) and was consistent with small fiber neuropathy in 25 of 48 (52.1%) cases, but Congo red staining was negative in all. Biopsies from other tissues (abdominal fat and bone marrow) were performed in two individuals and were also negative. Disorders that may cause or worsen polyneuropathy were identified in seven cases, including diabetes mellitus (T2D in two and type 1 in one case) and hyperglycemia in another four cases.

Echocardiography at a mean age of 42.4 ± 10.5 years (range 27–68) showed increased IVS thickness among 3 of 44 (6.8%) cases, two with the Ser77Tyr (with a negative skin biopsy, whereas scintigraphy was not conducted) and one with the Val122Ile variant. However, the GLS was normal in 18 cases, including one with Ser77Tyr and increased IVS thickness. CMR at a mean age of 59.8 ± 13.0 years (range 42–72) was normal in 5 of 6 cases, including the other Ser77Tyr case with increased IVS thickness. Scintigraphy was negative in all 18 cases who underwent the scan at a mean age of 49.8 ± 14.9 years (range 18–78), including a case with the Val122Ile variant and increased IVS thickness, attributed to a different monogenic cause.

### 3.4 TTR Variants in ES Databases

To assess the frequency of *TTR* variants in the Israeli population, we used two large ES databases from SMC and HMC. These included ES data of approximately 24.000 (HMC) and 12,620 (SMC), undertaken up to August 2024. At SMC, 2980 had full and 1940 partial Ashkenazy ancestry, 181 full and 521 partial Yemenite origin, 277 full and 972 partial Iraq origin, and 145 full and 351 partial Iranian origin (all Jewish). In total, 1191 were Arabs. In HMC, 18,565 were of Jewish and 5435 were of Arab ethnicity. In both databases, some Arab cases resided in the Palestinian Authority region.

We found 14 subjects with pathogenic *TTR* variants in these databases. The Ser77Tyr variant was identified in 4 subjects of Jewish Yemenite descent. Two were already under follow‐up in our clinic, and the others were a mother and a 5‐year‐old daughter who performed ES as part of an endocrine workup for the child. The mother was later examined in our clinic and included in the group of Ser77Tyr carriers.

Nine individuals, all of Muslim Arab origin, were detected with the Val122Ile variant. One was a 45‐year‐old female without ATTRv‐related symptoms who was later evaluated in our clinic and included in the carrier group. Data about the other eight individuals with this variant was unavailable, and we did not evaluate them clinically. The Val30Met variant was identified in another subject of Moroccan/Iraqi Jewish descent.

Three additional *TTR* missense variants were detected and classified as variants of uncertain significance (VUS). In the HMC ES database, the Ala109Thr (p.Ala129Thr) variant was found in two subjects, one Arab and the other Jewish, originating from central Asia. The Ser50Asn (p.Ser70Asn) variant was detected in a single individual of Arab origin. In the SMC ES database, the Arg103Cys (p.Arg123Cys) variant was observed in a single carrier of Arab origin. Although these variants were previously reported [29, 30], their interpretations in the ClinVar database are conflicting, and we consider them VUS according to the ACMG criteria.

In addition, we performed Sanger sequencing of Ser77Tyr and Val32Ala among self‐reported healthy individuals of Jewish Yemenite (*n* = 150) and Iranian (*n* = 100) descent, respectively. These variants were absent in all tested individuals. The Val30Met, Val122Ile, Ala81Val, and Glu89Ala variants were absent among 29,604 Ashkenazi Jewish individuals in the GnomAD database, version 4.1.0 [31].

## 4 Discussion

Previous studies about ATTRv amyloidosis in Israel focused on the Ser77Tyr variant, and less attention has been paid to other disease‐causing variants in *TTR*. In this study, we show that while most ATTRv amyloidosis cases in Israel are indeed caused by Ser77Tyr, other variants also contribute to the occurrence of this disease. Val122Ile was the second most frequent variant in general, and Thr60Ala was the second most common in ATTRv amyloidosis patients. Screening of ES databases indicated that *TTR* variants may be more common than previously estimated, mainly regarding Val122Ile.

Early detection of ATTRv amyloidosis is based on identifying typical “red flags” symptom clusters [32]. CTS is considered a common non‐specific finding in the general population but is also a frequent manifestation in ATTRv. Among our patient groups, it was the most frequent initial complaint of individuals with both neurologic and cardiac features. Thus, a high level of clinical suspicion for ATTRv amyloidosis is required in patients aged > 50 years with CTS accompanied by polyneuropathy or symptoms related to autonomic or cardiac dysfunction.

The diagnosis of ATTRv‐CM highly relies on cardiac scintigraphy, particularly in the absence of biopsy‐proven amyloid infiltration [16]. Similar to our previous report [12], we show here that the yield of scintigraphy for detecting ATTRv‐CM due to the Ser77Tyr variant was low even when typical cardiomyopathy was present. However, scintigraphy efficiently detected cardiac involvement in cases with the Thr60Ala and Val32Ala variants, even when the IVS thickness was not increased, particularly in Val32Ala patients. Therefore, the absence of increased IVS thickness or negative scintigraphy should not exclude the possibility of ATTRv amyloidosis in suspected cases [27]. CMR detection rate was high but was not diagnostic in all of our cases with ATTRv‐CM.

The *TTR* variants we identified have a global distribution. Ser77Tyr, recognized among the Jewish Yemenite population, is particularly frequent in ATTRv amyloidosis patients from northern France [33], with a similar predominantly neurologic phenotype. It was also found in other countries, such as Spain and the United States [34, 35]. The most frequently reported *TTR* variant worldwide is Val30Met [36] which was identified in three unrelated Jewish families of Ashkenazi and Tunisian origin in our sample, and a single subject from the ES databases. Val122Ile, which was the only variant identified in the Arab population, is found in up to 4% of African‐American individuals [37] and is thought to originate from West Africa [23]. Nevertheless, we also identified this variant in Jewish subjects (Ashkenazi and Iran/Yemen origin). However, based on ES data screening, it seems rare in the Jewish population and more prevalent in the Arab population. The Thr60Ala variant, which was detected in a family of Jewish Iraqi descent, is a common variant in the United Kingdom, particularly in Irish ancestry [38] and in the White population in the United States [35] with CTS and common autonomic and cardiovascular‐related symptoms as in our patients. The rare Val32Ala variant, identified in a Jewish family originating from Iran, was also previously described in a few patients [39, 40].

According to our presented data, we estimate the number of alive ATTRv‐amyloidosis patients in Israel in the range of 2–3 per million, but the carrier rate in the adult population is higher, which can be attributed to age‐dependent penetrance. Indeed, ATTRv amyloidosis is assumed to be more common than previously acknowledged, particularly in patients with idiopathic polyneuropathy and/or cardiomyopathy [41, 42, 43].

In our daily practice, we encourage adult family members, with and without ATTRv amyloidosis symptoms, to undergo genetic testing for the familial variant (cascade testing) to facilitate medical surveillance and early treatment for those at risk. Individuals found positive were included in this sample. Although additional patients and carriers may have declined testing, we estimate that their number is relatively small. Overall, we assume that the presented data renders a fair estimation of the most prevalent *TTR* variants in the Jewish and Arab populations in Israel. Some of the variants reported in the ES database are currently classified as VUS (Ala109Thr, Ser50Arg and Arg103Cys), but this classification may change in the future. Additionally, rare or even private variants will probably be recognized in the future as reporting disease‐causing variants in *TTR* is recommended according to ACMG guidelines for reporting of secondary findings in exome and genome sequencing data [44].

Interestingly, while males comprised approximately 50% of the overall sample, they were 71.4% of the patient population. This may suggest that males are more prone to develop ATTRv‐amyloidosis, consistent with previous reports [12, 34]. Nevertheless, we cannot exclude the possibility that females were less prone to pursue the appropriate medical evaluation or were not referred to our clinic for comprehensive clinical evaluation, which might have biased this finding. In more than half of the Ser77Tyr carriers, we found evidence of small fiber neuropathy, consistent with our previous report [12]. This was not identified in Thr60Ala carriers and was uncommon in Val30Met carriers, though the number of subjects in these groups was small.

Several other limitations should be acknowledged. First, the high prevalence of the Ser77Tyr variant may be biased, to some extent, as most of the carriers are family members of patients under our clinic's care who were screened due to their family history rather than clinical features. The specific familial and clinic networks may lead to over‐representation of certain variants and overshadow the contribution of additional rare variants. Second, some patients had additional systemic disorders that may cause or exacerbate neuropathy or cardiomyopathy (e.g., T2D or renal failure) which may affect the phenotype, and, actually, more than half of the patients with the Ser77Tyr variant had diabetes mellitus or prediabetes. Third, for histopathological diagnosis, we usually prefer employing a skin punch biopsy from the ankle, which has high sensitivity [45]. Biopsies from other tissues (e.g., endomyocard, fat, salivary glands, or sural nerve) were not routinely obtained. Thus, it is possible that a few variant carriers were wrongly not classified as ATTRv‐amyloidosis patients. Additional limitations include the cross‐sectional study design and lack of long‐term follow‐up for many carriers that did not have symptoms or findings during the initial evaluation.

To conclude, the presented data expand the knowledge about the ATTRv amyloidosis landscape in Israel, indicate the populations at risk, and suggest that the spectrum of *TTR* variants in the Israeli population is broader than previously assumed.

## Author Contributions

**Amir Dori:** conceptualization, data curation, formal analysis, funding acquisition, investigation, writing – original draft, writing – review and editing. **Odelia Chorin:** data curation, writing – review and editing, investigation. **Noa Ruhrman‐Shahar:** data curation, writing – review and editing, funding acquisition. **Avi Fellner:** data curation, writing – review and editing. **Tayir Alon:** data curation, writing – review and editing. **Haike Reznik‐Wolf:** data curation. **Ortal Barel:** data curation. **Dana Fourey:** data curation, writing – review and editing. **Osnat Itzhaki Ben Zadok:** data curation, writing – review and editing. **Yaron Aviv:** data curation, writing – review and editing. **Vera Nikitin:** data curation. **Merav Ben‐David:** data curation. **Efrat Shavit‐Stein:** formal analysis, writing – review and editing. **Rivka Goldis:** data curation. **Batia Kaplan:** data curation. **Daniela Shapiro:** data curation. **Elon Pras:** supervision. **Arthur Pollak:** data curation. **Vardiella Meiner:** data curation, writing – review and editing. **Michael Arad:** data curation, writing – review and editing. **Lior Greenbaum:** conceptualization, writing – original draft, writing – review and editing, formal analysis, investigation.

## Conflicts of Interest

Amir Dori received consultancy fees, honoraria for lectures and travel meeting attendance support, and investigator‐initiated research grant support from Pfizer, Alnylam Pharmaceuticals AstraZeneca, and Sanofi. Michael Arad received honoraria for lectures and fees for participation on an advisory board from Pfizer. Osnat Itzhaki Ben Zadok received honoraria for participation on an advisory board from Pfizer. Noa Ruhrman‐Shahar received fees for participation on an advisory board from AstraZeneca and investigator‐initiated research grant support from Pfizer.

## Supporting information


Data S1.

## Data Availability

The data that supports the findings of this study are available in the Supporting Information of this article.
