# Supplementary material for: Hereditary Transthyretin Amyloidosis in Israel: Genetic Landscape and Clinical Characteristics
Source: Eur J Neurol. 2025 Jan 29;32(2):e70057. doi: 10.1111/ene.70057 (PMC11775909; doi:10.1111/ene.70057)
Supplement: Supplementary file 1 — Data S1. [file ENE-32-e70057-s001.docx]

**Supplementary results - Detailed clinical evaluation according to disease-causing variants in *TTR***

**Ser77Tyr**

Among 23 patients with the Ser77Tyr (p.Ser97Tyr) variant, the most common initial symptoms were related to carpal tunnel syndrome (CTS), occurring in 12/23 (52.2%) of patients at a mean age of 58 ± 5.8 years (range 46-71). Other symptoms at an early stage were dyspnea in 4/23 (17.4%), sensory changes in the feet in 2/23 (8.7%), and erectile dysfunction occurring in 8/16 (50%) males. Polyneuropathy-related symptoms in the feet occurred at the age of 60 ± 5.8 years (range 51 - 71). At diagnosis of ATTRv amyloidosis (mean age of 62 ± 5.3 years, range 49–71), symptoms related to CTS remained most common, followed by sensory symptoms in the feet and dyspnea, and less frequent autonomic symptoms, lower extremity weakness, and gastrointestinal symptoms (Table 2).

At diagnosis, electrodiagnostic studies showed evidence for a large fiber sensory polyneuropathy in 16/23 (69.6%) of cases. Skin biopsy presented evidence for small fiber neuropathy in 17/19 (89.5%) of tested patients, with Congo red positive deposits in all of them. In two additional cases, a positive biopsy was collected from tissues other than the skin, and in another two individuals, a biopsy was not performed. Echocardiography demonstrated increased septal wall thickness in 18/23 (78.3%) and positive global longitudinal strain (GLS) in 14/16 (87.5%) of tested cases. Scintigraphy was positive in only 3/10 (30.0%), and in 4 negative cases, echocardiography showed increased septal wall thickness and positive GLS. Cardiac MRI (CMR) was positive in 11/12 (91.7%) of patients who underwent testing.

As the early symptoms were commonly neuropathy and autonomic-related, and some of the patients showed no cardiac involvement, the Ser77Tyr variant was considered to exhibit a predominantly neurologic phenotype.

Interestingly, two carriers had evidence of amyloid and small fiber neuropathy on skin biopsy but had no clinical complaints. Both were treated with Tafamidis. Upon follow-up, one remained with no symptoms for more than five years, and the other developed amyloid cardiomyopathy.

Forty-five carriers of the Ser77Tyr variant (19 males, 42.2%) did not meet the criteria for diagnosing amyloidosis. Various sensory symptoms in the hands were reported by 21/45 (46.7%) and in the feet by 10/45 (22.2%) individuals. A skin biopsy was performed in 40 of them at a mean age of 41.4 ± 11.3 years (range 22– 69), and in one patient an additional abdominal fat pad biopsy was obtained. Congo red staining was negative in all, but 23/40 (57.5%) showed a low epidermal nerve fiber density (ENFD) consistent with small fiber neuropathy. Electrodiagnostic studies were performed at approximately the same time and showed no evidence for a large-fiber polyneuropathy, but eight subjects showed supportive evidence for a CTS and in five the sympathetic skin response (SSR) was abnormal. Echocardiography at age 43.0 ± 11.0 years (range 27–69) showed a mildly increased IVS thickness of 12 mm in 2/32 carriers, but in both cases skin biopsy was negative and CMR was normal. In two additional cases CMR was negative. Overall, scintigraphy was negative in 8 carriers at a mean age of 50.2 ± 12.3 years (range 27 – 68). In three cases, both skin biopsy and cardiac evaluation were not completed.

One adult Ser77Tyr carrier was introduced to our clinic following detection by the exome sequencing (ES) database screening. This was a 41-year-old female who had no symptoms of ATTRv amyloidosis and had normal electrodiagnostic and echocardiography studies. Her skin biopsy showed evidence of small fiber neuropathy without Congo red positive deposits. Accordingly, she was included among the group of Ser77Tyr carriers.

**Thr60Ala**

The Initial symptoms related to the Thr60Ala (p.Thr80Ala) variant were CTS in all five patients, which occurred at age 52.9 ± 6.2 years (range: 43–60). Interestingly, two of the males were identical twins, and both developed CTS symptoms at the same age (52.6 years). This was followed by dyspnea in 4/5 (80%) cases and autonomic and sensory symptoms related to polyneuropathy in 3/5 (60%). Cardiac-related symptoms occurred prior to polyneuropathy/autonomic symptoms in only one case, simultaneously or following these in three cases, and were absent in one.

At diagnosis (age 61.0 ± 12.2 years, range 43–75), large fiber polyneuropathy was evident in only one patient, small fiber neuropathy in 2/4 (50%), and Congo red-positive deposits in the skin in 3/4 (75%) cases.

Echocardiography showed increased IVS thickness in 3/5 (60%), scintigraphy was positive in 4/5 (80%), and in one case, CMR showed evidence for cardiomyopathy while scintigraphy was negative (Perugini score of 1). Spinal stenosis was significant in 3/5 (60/%) cases, with surgery required in one.

As cardiac involvement was positive in all cases and polyneuropathy symptoms and signs were present in only some, the Thr60Ala variant was considered to exhibit a predominantly cardiac phenotype.

Two additional subjects, a 30-year-old male and a 41-year-old female, had no relevant symptoms. The male showed bilateral median neuropathy at the wrist on NCSs, but otherwise, their electrodiagnostic studies were normal. Their skin biopsy showed normal ENFD and was negative for Congo red staining, and echocardiography showed no evidence for increased IVS thickness. The female had a negative scintigraphy study as well. Accordingly, these were considered carriers of the *TTR* Thr60Ala variant.

**Val32Ala**

In all three patients with the Val32Ala (p.Val52Ala) variant, the initial symptoms were related to CTS, occurring at the age of 57.2 ± 5.7 years (range 52–63), followed by sensory symptoms in the feet at an average age of 58.2 ± 4.8 years (range 54-63). Two patients had autonomic symptoms and lower extremity weakness, and a single patient developed dyspnea and gastrointestinal symptoms.

At diagnosis (age 59.9 ± 3.8 years, range 56–64), only one patient had large-fiber sensory polyneuropathy. Skin biopsy showed small fiber neuropathy in two males (67%), with Congo red positive deposits in both cases. One female patient had no evidence of small fiber loss nor amyloid deposits in the skin.

Echocardiography showed normal IVS thickness but abnormal scintigraphy in all 3 cases. CMR was performed and found negative in one of these cases. The female patient had an abnormal GLS and end-diastolic ejection fraction of 29% and an endomyocardial biopsy was performed, demonstrating positive Congo-red deposits.

The p.Val32Ala variant, therefore, exhibited a mixed neurologic and cardiac phenotype.

An additional female carrier of this variant was examined at the age of 60 years. She was asymptomatic, had normal scintigraphy but no other documented evaluation, and was lost for follow-up.

**Other variants**

Only a single subject with the Val122Ile (p.Val142Ile) and the Val30Met (p.Val50Met) variant each, were considered as ATTRv-amyloidosis patients.

The patient with Val122Ile variant was diagnosed at the age of 75 years. He had unilateral CTS symptoms 20 years earlier, and at diagnosis, these were bilateral, accompanied by polyneuropathy-related symptoms in the feet. He complained of dyspnea, his IVS diameter was 12 mm, and the diagnosis was confirmed by positive scintigraphy and CMR.

All eight additional carriers of Val122Ile were younger (range 18–54 years) and were not expected to express ATTRv-amyloidosis at that age. In one case, the IVS diameter was severely enlarged (27 mm), and the CMR was abnormal. However, scintigraphy was negative, and therefore, the hypertrophy was attributed to a co-occurring homozygous *TNNI3* c.434G>A (p.Arg145Gln) pathogenic variant. None of these carriers performed a tissue biopsy.

A single male patient with the Val30Met variant had bilateral CTS and sensory-motor polyneuropathy symptoms at the age of 72 years, confirmed within a year by electrodiagnostic studies. He had symptoms of orthostatism but no dyspnea. Echocardiography showed an IVS diameter of 15 mm, and a skin biopsy confirmed amyloid infiltration, with Congo red positive deposits in addition to small fiber neuropathy at age 72.5 years. Additional four carriers (one male) harboring the Val30Met variant were followed at the clinic, and at a mean age of 61.5 ± 14.7 years (range 48–77) none had clinical, electrodiagnostic, echocardiographic or skin biopsy evidence of amyloidosis.

A single female patient had the Ala81Val (p.Ala101Val) variant and presented at age 54 years with bilateral CTS and a year later with progressive dyspnea. At age 63 years, her echocardiography showed an IVS diameter of 17 mm, GSL of -9.2, and positive CMR. Abdominal fat and endocard biopsies were negative, but a skin biopsy was positive for Congo-red staining, confirming the diagnosis of amyloidosis. NCS showed evidence for bilateral CTS, without large fiber polyneuropathy. Skin biopsy demonstrated evidence for a small fiber polyneuropathy.

Last, another male patient harbored the Glu89Val (p.Glu109Val) variant and presented with dyspnea, with onset at age 50 years. At diagnosis, at age 65, echocardiography showed an IVS diameter of 23 mm, end-diastolic ejection fraction of 45%, and positive scintigraphy. He had no neurologic symptoms.
